# Mitochondrial uncoupling, energy substrate utilization, and brown adipose tissue as therapeutic targets in cancer

**DOI:** 10.1038/s44324-025-00080-3

**Published:** 2025-09-22

**Authors:** Maurizio Ragni, Chiara Ruocco, Enzo Nisoli

**Affiliations:** Center for Study and Research on Obesity, Department of Medical Biotechnology and Translational Medicine, University of Milan, Via Vanvitelli 32, 20129 Milan, Italy

**Keywords:** Endocrine system and metabolic diseases, Metabolism, Cancer

## Abstract

Mitochondria play a central role in regulating cellular energy metabolism, redox homeostasis, and biosynthesis. Mitochondrial uncoupling, through the alteration in the permeability of the inner mitochondrial membrane (IMM) to the leak of protons without adenosine triphosphate (ATP) synthesis, regulates thermogenesis, glucose and lipid metabolism, and reactive oxygen species (ROS) generation. In brown adipose tissue (BAT), proton leak via uncoupling protein 1 (UCP1) is essential for thermogenesis and has been shown to improve systemic glucose homeostasis, and recent studies indicate that BAT activation can also suppress tumor growth by competing with cancer cells for glucose. Several small-molecule mitochondrial uncouplers have demonstrated anticancer effects in preclinical models, although endogenous UCPs—particularly UCP2—are often upregulated in tumors, where they may support tumor growth by buffering ROS and increasing metabolic flexibility. These seemingly contradictory observations highlight the context-dependent effects of mitochondrial uncoupling in cancer. Here, we review current understanding of mitochondrial uncoupling mechanisms, the roles of UCP isoforms, and the metabolic interplay between BAT, cancer cells, and the tumor microenvironment, with a focus on therapeutic implications.

## Introduction

Life requires energy, and cells extract this energy from nutrients, storing it in ATP to fuel biological processes. While macronutrients such as lipids, amino acids, and glucose can all be used for energy production, only glucose can be metabolized anaerobically, i.e., without oxygen. Glycolysis, the anaerobic breakdown of glucose into lactate, yields only 2 ATP molecules per mole of glucose, whereas mitochondrial oxidation of pyruvate produces 36 ATP molecules, and this adaptation likely provided an evolutionary advantage during the transition from an oxygen-poor to an oxygen-rich atmosphere. The ability to maximize energy extraction from glucose also offered a significant advantage in the nutrient-scarce environments where early life evolved. However, the current overabundance of food and sedentary lifestyle continuously challenge the proper mitochondrial handling of energy substrates, leading to nutrient overload and to metabolic inflexibility, which underlies metabolic disorders such as obesity, diabetes and cardiovascular diseases. In this context, it is not surprising that cancers also display several metabolic alterations, among which the engagement in anaerobic glycolysis despite the presence of oxygen (i.e., the Warburg effect) and the reprogramming of mitochondrial metabolism are the most important^1^.

**Fig. 1:**
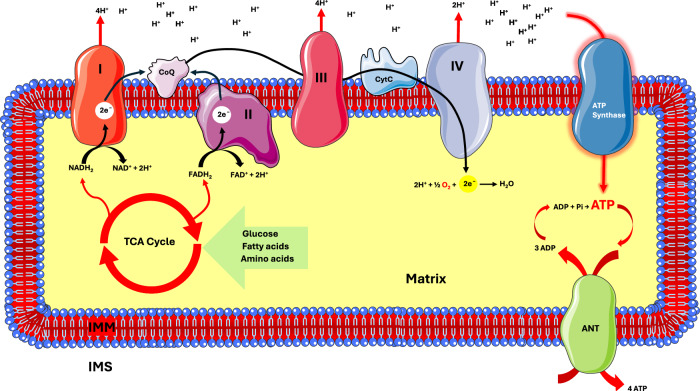
Mitochondrial oxidative phosphorylation. Nutrients are oxidized in mitochondrial matrix to generate a transmembrane electrochemical gradient, which is used for ATP synthesis, whose thermodynamic driving is maintained by ANT. Complexes I and II oxidize NADH and FADH_2_, respectively, which are generated by the oxidation of energy substrates in the tricarboxylic acid (TCA) cycle. Electrons (2e^-^) are transferred along the electron transport chain (ETC), due to increasing redox potentials of mitochondrial complexes. The energy released is used by Complexes I, III, and IV to pump protons into the intermembrane space (IMS), generating a proton gradient—also known as the protonmotive force—across the inner mitochondrial membrane. Energy derived from re-entry of protons into the matrix via ATP synthase (Complex V) drives ATP synthesis. The adenine nucleotide translocase (ANT) facilitates the exchange of matrix-derived ATP for cytosolic ADP, maintaining the ATP synthesis reaction far from thermodynamic equilibrium. IMS: intermembrane space. See main text for additional abbreviations. Image adapted from Servier Medical Art (https://smart.servier.com/), licensed under CC BY 4.0 (https://creativecommons.org/licenses/by/4.0/).

In glycolysis, ATP is generated through substrate-level phosphorylation, where oxidation of an intermediate is directly coupled to ATP production. In contrast, mitochondrial oxidative phosphorylation (OXPHOS) couples ATP synthesis to the generation of a protonmotive force (Δp), driven by the electron transport chain (ETC) complexes in the IMM (Fig. 1). Thus, the mitochondrial membrane plays a crucial role in energy metabolism; its transport systems and permeability to protons are key regulators of energy production (i.e., energy generation from nutrient substrates, energy expenditure (EE) through thermogenesis, the adaptation to environmental or nutritional stimuli) and metabolic flexibility—the capacity of cells and organisms to switch between different nutrient sources. Noteworthy, cancer cells display a hyperpolarised mitochondrial membrane potential (i.e. more negative) as compared to normal cells, which characterizes the transition to the neoplastic state and is directly related to an high glycolytic activity and a low OXPHOS rate in tumors^2,3^.

The chemiosmotic theory implies that the efficiency of ATP production can be modulated through changes in membrane permeability^4^. In response to environmental or nutritional stimuli, mammals have developed mechanisms to waste energy, promoting inefficient ATP synthesis and energy dissipation through processes like proton leak and BAT thermogenesis. These processes increase the IMM’s permeability to protons, resulting in what is often referred to as basal and inducible proton leak, respectively. Inducible BAT thermogenesis is activated by sympathetic nervous system (SNS) output in response to environmental stimuli such as cold exposure and overnutrition. However, while cold adaptation clearly explains the evolutionary advantage of dissipating energy as heat, it is less obvious why organisms would have evolved energy-wasting mechanisms, such as diet-induced thermogenesis (DIT), in response to food intake. This is particularly puzzling given that famine, not abundance, has been the primary evolutionary pressure, favoring energy storage via insulin signaling and promoting reproductive success in organisms characterized by a low EE. Evidence in mice has both supported and refuted the existence of DIT, and the role of BAT in humans, although implicated in both cold-induced thermogenesis (CIT) and DIT, seems to be more strongly linked to glucose homeostasis and cardiometabolic health.

Interestingly, recent studies in mice suggest that activated BAT may also serve as an anti-cancer mechanism^5,6^, potentially opening new therapeutic avenues for human health. In addition, several chemical (i.e. “exogenous”) mitochondrial uncouplers show antitumoral properties, and some of them are currently being tested in clinical trials^7^.

In this review, we will discuss the connection between mitochondrial membrane potential, its role in the regulation of EE, thermogenesis and energy substrates handling through uncoupling of OXPHOS, and the possible outcomes on cancer therapy.

## Mechanisms and physiological roles of mitochondrial proton leak

### Fundamentals of proton leak

In aerobic eukaryotic cells, mitochondrial OXPHOS extracts the energy stored in nutrients to produce ATP through a series of redox reactions. Within the tricarboxylic acid (TCA) cycle, reduced substrates are oxidized, generating electron donors—nicotinamide adenine dinucleotide (NADH) and flavin adenine dinucleotide (FADH_2_). These carriers transfer high-energy electrons to the ETC, which comprises four major complexes: complex I (NADH: ubiquinone oxidoreductase, CI), complex II (succinate dehydrogenase, CII), complex III (ubiquinol-cytochrome c reductase, CIII), and complex IV (cytochrome c oxidase, CIV). Complexes I and II accept electrons from NADH and FADH_2_, respectively, and transfer them to coenzyme Q (CoQ), which acts as a mobile electron carrier between CI/CII and CIII. Electrons are subsequently transferred via cytochrome c (cytC) to CIV, where molecular oxygen (O_2_) is the final electron acceptor, being reduced to water (H_2_O). The energy released during this electron transfer drives the active translocation of protons (H^+^) across the IMM by complexes I, II, and IV. This proton pumping generates an electrochemical gradient known as the protonmotive force (Δp), which comprises an electrical potential (ΔΨ) and a transmembrane pH gradient (ΔpH). The energy stored in this gradient is then utilized by the F_O/_F_1_ ATP synthase (complex V) to drive ATP synthesis, coupling the return of protons to the matrix with the phosphorylation of ADP to ATP (ADP + P_i_ → ATP) (Fig. 1).

To ensure that ATP hydrolysis remains sufficiently exergonic to power cellular processes, cells maintain a high ATP/ADP ratio, thereby keeping the reaction ADP + Pi → ATP far from thermodynamic equilibrium. As a result, mitochondrial OXPHOS is tightly regulated by cellular energy demand and ATP consumption. When ATP consumption increases, the ATP/ADP ratio decreases, signaling an energy deficit. This stimulates the activity of complex V, leading to a reduction in Δp. To compensate, electron transport and substrate oxidation are upregulated to restore Δp and sustain ATP production. Conversely, when the ATP/ADP ratio is high—indicating energy sufficiency—proton flux through complex V diminishes, Δp increases, and mitochondrial respiration slows accordingly. This feedback regulation is also evident in isolated mitochondria: the addition of ADP triggers a rapid increase in oxygen consumption rate (OCR), underscoring the tight coupling between substrate oxidation, oxygen consumption, and ATP synthesis.

**Fig. 2:**
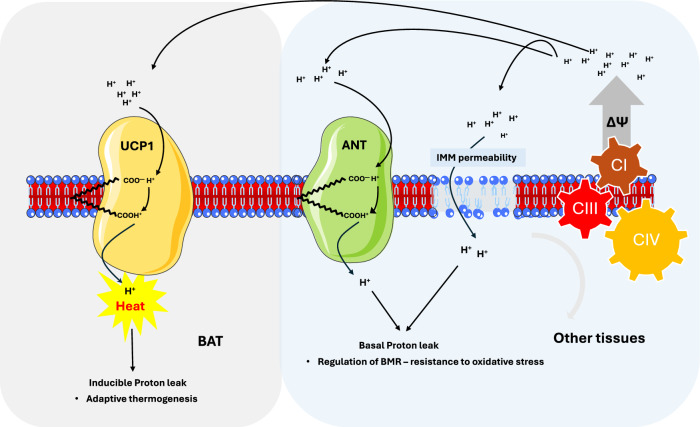
Mechanisms of mitochondrial proton leak. Basal and inducible mitochondrial proton leak are involved in distinct processes of cell bioenergetic, regulating BMR/oxidative stress or BAT thermogenesis, respectively. Inducible proton leak is catalyzed by UCP1 in BAT, resulting in heat production. In contrast, basal proton leak is primarily mediated by ANT in non-BAT tissues. One model suggests that both inducible and basal proton leak are both protein-mediated processes that share a common mechanism involving free fatty acids (FFAs) as essential cofactors and proton carriers. An alternative model proposes that basal proton leak arises from increased permeability of the inner mitochondrial membrane (IMM), driven by changes in its phospholipid composition. In all cases, dissipation of the mitochondrial membrane potential (Δψ) leads to increased O_2_ consumption and energy expenditure to restore Δψ. This mechanism underlies adaptive thermogenesis (via UCP1), and may also contribute to basal metabolic rate (via ANT or membrane composition) and oxidative stress resistance (via “mild” uncoupling). See main text for abbreviations. Image adapted from Servier Medical Art (https://smart.servier.com/), licensed under CC BY 4.0 (https://creativecommons.org/licenses/by/4.0/).

However, the coupling between substrate oxidation and ATP synthesis is not absolute. Protons can leak back into the matrix, independently of ATP synthase, through a process known as proton leak (proton leak), thereby dissipating the Δp without generating ATP (Fig. 2). This uncoupled proton influx leads to depolarization of IMM, resulting in energy loss as heat. To restore ΔΨ, mitochondria increase substrate oxidation and OCR, ultimately elevating EE. Proton leak can be experimentally detected in vitro by measuring sustained mitochondrial respiration and a decline in ΔΨ in the presence of ATP synthase inhibitors such as oligomycin, which blocks ATP production. By modulating IMM proton conductance, proton leak serves as a regulatory mechanism for metabolic rate, thermogenesis, and overall EE. The magnitude and physiological relevance of this process vary across tissues and are influenced by environmental and hormonal factors. Importantly, proton leak is now recognized as an intrinsic property of mitochondria, rather than merely a byproduct of inefficiency.

### Proton leak and the regulation of metabolic rate and reactive oxygen species

In mammals, proton leak exists in two forms: an inducible, protein- and free fatty acids (FFA)-mediated proton leak—central to BAT thermogenesis—and a basal, often referred to as constitutive, proton leak that occurs in mitochondria across various tissues and cell types.

While inducible proton leak is a well-characterized process underlying BAT thermogenesis (see below), the nature and the physiological function of basal proton leak remain less well understood. Nevertheless, its impact on mitochondrial function has led to the hypothesis that basal proton leak contributes significantly to the regulation of basal metabolic rate (BMR), and several experimental studies support this hypothesis. In rat liver mitochondria, basal proton leak is estimated to account for approximately 20-25% of BMR. In skeletal muscle, a highly metabolically active tissue, it may contribute to up to 50% of mitochondrial respiration. Other cell types also exhibit variable degrees of proton leak: for example, thymocytes and neurons display intermediate leak levels, whereas the INS-1E insulinoma cell line exhibits an exceptionally high rate of respiration not coupled to ATP synthesis, with up to 70% of total oxygen consumption being attributed to proton leak^8–12^.

Given the liver’s high metabolic activity and the substantial mass of skeletal muscle, basal proton leak is considered a key contributor to whole-body BMR. Supporting this view, basal proton leak is modulated by thyroid hormones and correlates with thyroid status: it is elevated in hyperthyroid rats and reduced in hypothyroid animals compared to euthyroid controls^13^. Furthermore, the age-related decrease in circulating tri-iodothyronine (T3) levels is associated with decreased proton leak in skeletal muscle mitochondria^14,15^. Despite its physiological relevance, the mechanistic basis of basal proton leak remains under debate, and it is unclear whether basal proton leak is an intrinsic property of the IMM or whether it requires the involvement of specific proteins or FFAs. Some studies suggest a relationship between basal proton leak and the biophysical characteristics of the IMM, including its phospholipid composition and surface area^16–18^. In particular, the reduced proton leak in hypothyroid rats has been associated with altered IMM phospholipid composition, notably a decreased polyunsaturated phospholipid n-6/n-3 ratio^19^ (Fig. 2). However, this hypothesis is not universally supported, since other studies report that changes in FFA unsaturation have only a minor effect on proton leak, accounting for approximately 5% of total proton conductance^20^. Instead, accumulating evidence points to the mitochondrial carrier adenine nucleotide translocase (ANT) as a principal mediator of constitutive proton leak, a hypothesis discussed in detail in the following section.

Another major proposed function of basal proton leak is the regulation of oxidative stress and the production of reactive oxygen species (ROS). The proton circuit across the IMM can be viewed as an electrical circuit, where proton flow (I) corresponds to current, the Δp to voltage (V), and IMM permeability to resistance. According to Ohm’s law, if resistance remains constant, Δp should increase linearly with proton current. However, in the presence of proton leak, increased membrane conductance acts like a short circuit, preventing excessive Δp buildup. While this analogy is conceptually useful, experimental data from isolated mitochondria clearly reveal that proton leak displays non-ohmic behavior. Simultaneous measurements of respiration rate and membrane potential in the presence of ATP synthase inhibition (e.g., by oligomycin) reveal that proton leak becomes significantly more pronounced at high ΔΨ—typically above ~150 mV in liver mitochondria—and is negligible at lower ΔΨ. This voltage-dependent behavior implies that proton leak is minimal when ATP synthesis is active and Δp is low, thereby ensuring efficient energy coupling. Conversely, under conditions of high ΔΨ and low ATP demand—such as during nutrient excess or low workload—proton leak increases and serves a protective function. In such states, the highly reduced ubiquinone (CoQ) pool and accumulation of metabolic intermediates at complex I can promote reverse electron transfer (RET) from ubiquinol to NAD^+^, leading to the partial reduction of O_2_ and generation of superoxide anion (O_2_•^−^)^21^. Thus, basal proton leak may function as a “safety valve” to limit oxidative damage by preventing excessive buildup of Δp, thereby avoiding over-reduction of ETC and minimizing superoxide generation^22^.

According to the free radical theory of aging, increased ROS production contributes to the aging process. In this context, proton leak-mediated “mild uncoupling” of OXPHOS has been proposed as a mechanism to limit ROS accumulation, thereby mitigating age-related oxidative damage. Supporting this hypothesis, studies in outbred mice have shown that long-lived individuals exhibit higher metabolic rates, increased oxygen consumption, and elevated proton leak rates in skeletal muscle mitochondria. Moreover, pharmacological uncoupling with low doses of agents such as 2,4-dinitrophenol (DNP) has been reported to extend lifespan and reduce ROS levels in mice^23,24^. However, the “mild uncoupling” hypothesis remains controversial. For example, significant RET—a major source of mitochondrial ROS—typically requires high concentrations of succinate, which may not occur under physiological conditions^25^. Additionally, in *C. elegans*, long-lived *daf-2* mutants exhibit enhanced mitochondrial efficiency rather than increased uncoupling^26^. Further challenging the hypothesis, studies in human fibroblasts and yeast have shown that low-dose uncoupling with agents like carbonyl cyanide-p-trifluoromethoxy-phenylhydrazone (FCCP) or DNP can promote premature senescence^27^.

### Inducible and basal proton leak mediated by UCP1 and ANT

In contrast to basal proton leak, inducible proton leak is a well-characterized physiological process which underlies the thermogenic function of BAT. In this specialized tissue, the mitochondrial carrier UCP1enables non-shivering thermogenesis by dissipating the proton gradient across the IMM, releasing energy as heat. However, in tissues other than BAT, up to two-thirds of basal proton leak is attributed to the activity of another mitochondrial carrier: adenine nucleotide translocase (ANT). ANT exchanges mitochondrial ATP with cytosolic ADP, thereby supplying the substrates necessary for ATP synthase activity (Figs. 1 and 2). Beyond its canonical transport role, ANT exhibits protonophore activity in both liver and heart mitochondria, and basal proton leak has been found to correlate positively with ANT protein level^28–30^.

Both UCP1 and ANT require FFAs as essential cofactors for their uncoupling activity. This requirement provides a mechanistic basis for the long-recognized role of FFAs in regulating mitochondrial proton conductance across various tissues^31^ (Fig. 2). The hypothesis that ANT, like UCP1, can mediate FFA-dependent proton translocation was first proposed in 1988^32^ and has been experimentally validated since then. The uncoupling effect of palmitate in skeletal muscle and liver mitochondria is abrogated by ANT inhibitors such as carboxyatractylate and bongkrekic acid^31^; additionally, a non-metabolizable long-chain fatty acid analogue has been shown to induce mitochondrial uncoupling in isolated liver cells, an effect that is partially inhibited by atractyloside^33^. Furthermore, in skeletal muscle mitochondria, carboxyatractylate-sensitive proton leak is abolished by GDP, further supporting ANT’s role in mediating proton leak^34^.

The involvement of ANT in thyroid hormones (TH) -mediated regulation of cell metabolism is also well established. In rat liver, the transition from a hypothyroid to a hyperthyroid state is associated with increased oligomycin-sensitive oxygen consumption and a higher cytosolic ATP/ADP ratio, and TH increase ANT expression across multiple tissues^35^, an effect that correlates with enhanced mitochondrial uncoupling^36^. More recent studies using patch-clamp techniques on isolated mitoplasts have confirmed that ANT mediates FFA-dependent proton leak in tissues beyond BAT, through a mechanism analogous to that of UCP1 in BAT thermogenesis^37^. Remarkably, ANT and UCP1 show reciprocal inhibition, suggesting that ANT primarily supports constitutive mitochondrial uncoupling in tissues with high ATP turnover, whereas UCP1 mediates inducible uncoupling in BAT^37^.

Interestingly, chemical uncouplers like DNP, FCCP, and 2-fluorophenyl){6-[(2-fluorophenyl)amino](1,2,5-oxadiazolo [3,4-e]pyrazin-5-yl)} amine (BAM15) have also been reported to activate ANT and UCP1, suggesting that their mechanisms of action may have been previously misunderstood^38^. However, this interpretation has been challenged by recent findings showing no difference in sensitivity to FCCP and DNP between mitochondria from UCP1-knockout and wild-type mice^39^. These discrepancies may reflect differences in experimental systems, such as the use of isolated mitochondria versus mitoplast preparations.

ANT is among the most abundant mitochondrial proteins, accounting for approximately 10% of total mitochondrial protein content. In contrast to UCP homologues, which exhibit tissue-specific expression, ANT is ubiquitously expressed, making it a key candidate for regulating whole-body basal proton leak. Nevertheless, whether ANT activity directly contributes to BMR or overall EE remains uncertain. Studies using ANT-overexpressing or ANT-deficient mouse models have yielded conflicting results. For instance, ANT1-knockout mice—lacking the isoform predominantly expressed in heart, skeletal muscle, and brain—are protected against high-fat diet (HFD)-induced metabolic dysfunction and exhibit increased mitochondrial uncoupling^40^. Similarly, liver-specific ANT2 knockout mice display a lean phenotype and are resistant to hepatic steatosis, obesity, and insulin resistance when fed a lipogenic diet, together with elevated uncoupled respiration^41^. Myeloid-specific ANT2 knockout mice show reduced adipose tissue inflammation and improved glucose tolerance under HFD, although mitochondrial respiration remains unchanged between genotypes^42^. Conversely, ANT1 overexpression has been shown to confer cardioprotection in heart disease models, with variable effects on mitochondrial respiration, oxidative stress, and apoptosis^43–45^. Despite these findings, functional data on ANT in metabolically relevant tissues such as white and BAT are still lacking, limiting our understanding of its role in systemic energy homeostasis. Further research is also needed to clarify the contributions of the different ANT isoforms (ANT1-4), which may have overlapping but distinct functions, and to account for ANT’s central role in ADP/ATP exchange, a process critical for mitochondrial metabolism.

Box 1 Alternative thermogenic mechanisms in brown or white adipocytes Beyond ANT-mediated proton leak and sarcolipin-dependent ATP cycling in muscle, UCP1-independent thermogenic processes have also been described in BAT and beige fat.

- *Futile creatine cycle (FCC)*. In beige fat mitochondria, addition of creatine drives respiration under limiting ADP conditions (state 4), suggesting an excess ADP production via creatine kinase (CK), increasing OCR through cycling of ATP synthase, ANT, and CK^194^. In vivo, β-GPA-mediated creatine depletion reduces β-adrenergic-induced OCR in iWAT and BAT and blunts the temperature rise of cold-exposed UCP1–/– mice^194^. Deletion of creatine biosynthetic enzymes (CKB, TNAP, GATM) reduces CL316,243-induced EE and impairs thermogenesis, which is restored by creatine supplementation^195–197^. However, criticisms have been raised toward FCC, with regard to transient mitochondrial effects, TNAP specificity and low ATP synthase activity in BAT^198^; nevertheless, re-expression of CKB in UCP1/CKB-deficient adipocytes restores FCC, supporting the existence of UCP1-independent thermogenesis via ATP hydrolysis^199^.
- *Calcium cycling*. In beige adipocytes with low UCP1 and high ATP synthase expression, noradrenaline stimulates Ca²⁺ release from ER stores via RyR2, which activates mitochondrial PDH and is recycled by SERCA2b, forming a futile ATP-consuming cycle^200^. Overexpression of Prdm16 in UCP1–/– mice enhances EE and thermogenesis under cold and HFD conditions, while RyR2 and SERCA2 modulate glucose homeostasis at thermoneutrality^200^.
- *Futile lipid cycles*. In rat WAT, ~30% of FFAs released during fasting are re-incorporated into TAGs, and ~60% in humans across WAT, muscle, and liver^201,202^. This process is fueled by ATP-consuming reactions involving glycerol kinase (GyK), expressed in BAT and by PEPCK-C–mediated glyceroneogenesis in WAT^203,204^. Cold exposure induces genes of both fatty acid synthesis and oxidation in BAT^205–207^ while WAT also shows cold-induced FFA/TG cycling (without UCP1 involvement), more pronounced in obesity-resistant A/J than in B6 mice^208^. While its role in glucose homeostasis is well supported^209^, the quantitative contribution of this cycle to energy balance remains uncertain.

## Thermogenesis in humans and mice: evolutionary perspectives and therapeutic approaches

### Muscle and BAT thermogenesis

Endogenous heat production in animals includes both obligatory and adaptive (facultative) thermogenesis. Obligatory thermogenesis refers to the heat generated by basal metabolic processes under thermoneutral conditions, whereas adaptive thermogenesis is an acute response to cold that includes both shivering and non-shivering thermogenesis (NST). In most vertebrates, including humans, the primary thermogenic organ is skeletal muscle, where heat is generated during shivering and contraction through myosin-driven ATP hydrolysis and Ca^2+^ transport by the Sarco-Endoplasmic Reticulum Calcium ATPase (SERCA) pump. However, because sustained shivering is energetically costly and behaviorally disruptive, non-shivering mechanisms within muscle are also engaged to maintain thermal homeostasis. One such mechanism involves sarcolipin—a small regulatory peptide that uncouples SERCA activity from ATP hydrolysis—promoting futile cycling of Ca^2+^ uptake and release, thereby producing heat without inducing muscle contraction^46^.

In endothermic animals, BAT represents an additional major site of NST, where UCP1 enables proton leak across the IMM, thereby bypassing ATP synthesis and dissipating the electrochemical gradient as heat (Fig. 2). This UCP1-mediated uncoupling allows for the rapid conversion of stored energy into thermal energy. The expression and activity of UCP1 are tightly regulated by environmental and nutritional cues, particularly in response to cold exposure or overfeeding, through activation of the sympathetic nervous system and norepinephrine release.

Both BAT and skeletal muscle play essential roles in thermogenesis in higher mammals. This functional convergence is supported by their shared developmental origin from common myogenic progenitor cells, in contrast to the distinct mesenchymal lineage that gives rise to white adipocytes^47^. BAT also expresses several muscle-associated proteins related to mitochondrial activity and lipid metabolism, including muscle-type carnitine palmitoyl transferase I (M-CPT I), which regulates mitochondrial ß-oxidation, and heart-type fatty acid-binding protein (H-FABP), which regulates fatty acids (FA) trafficking^48^. Additionally, UCP3—a member of the UCP family expressed predominantly in skeletal muscle and brown adipocytes—contributes to mitochondrial FA export during lipid overload^49^. Cold exposure also induces the expression of myoglobin in BAT, paralleling its role in muscle as an oxygen storage and delivery molecule^50^, underscoring a shared adaptive mechanism between these two thermogenic tissues.

This evolutionary and functional relationship between BAT and skeletal muscle is further reinforced by inter-tissue communication through secreted factors—batokines from BAT and myokines or exerkines from muscle—that modulate each other’s activity. For instance, prolonged exercise promotes the release of apelin, β-aminoisobutyric acid (BAIBA), FNDC5 (the precursor of *irisin*), and lactate, all of which enhance cardiometabolic health by activating BAT or promoting the browning of white adipose tissue (WAT). Conversely, BAT secretes lipid-derived batokines such as 12,13-dihydroxy-9Z-octadecenoic acid (12,13-diHOME), which enhances FA uptake and oxidation in skeletal muscle^51–55^, highlighting a bidirectional regulatory axis between these thermogenic tissues.

Amino acids (AAs) are essential metabolic fuels for skeletal muscle, supporting protein synthesis during exercise and serving as substrates for mitochondrial energy production. In line with the emerging crosstalk between muscle and BAT, essential AAs (EAAs) have also been identified as thermogenic fuels for BAT. Dietary EAA supplementation has been shown to activate BAT thermogenesis and promote UCP1-dependent mitochondrial uncoupling, thereby conferring protection against obesity and improving insulin sensitivity^56^. Interestingly, EAA-enriched diets also ameliorates cardiac hypertrophy and suppress tumor growth in mouse models^57,58^. However, the extent to which these systemic benefits are mediated by BAT activation remains to be elucidated.

### Therapeutic potential of BAT activation

**Fig. 3:**
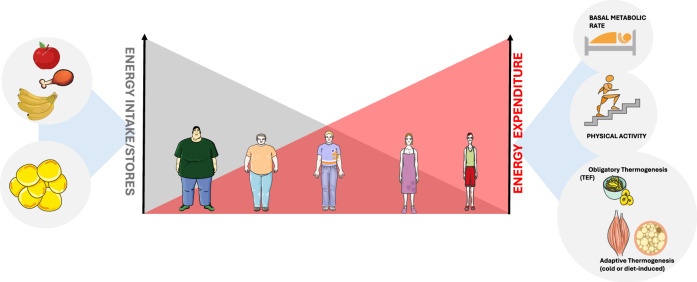
Regulation of body weight by energy balance. Body weight is determined by the balance between energy input and energy output. Input includes food intake or energy stores in white adipose tissue (WAT), while output is set by total energy expenditure (TEE), which comprises basal metabolic rate (BMR), physical activity, and thermogenesis—both obligatory and adaptive. Obligatory thermogenesis refers to the thermic effect of food, while adaptive thermogenesis includes both cold-induced and diet-induced thermogenesis. A sustained negative energy balance—where energy expenditure exceeds intake—lead to weight loss. Conversely, excessive energy intake not balanced by increased expenditure results in body weight gain. Image adapted from Servier Medical Art (https://smart.servier.com/), licensed under CC BY 4.0 (https://creativecommons.org/licenses/by/4.0/).

The discovery of metabolically active BAT in adult humans has renewed interest in targeting BAT as a therapeutic strategy for obesity and type 2 diabetes (T2DM). Body weight (BW) is determined by the balance between energy intake and total EE (TEE), which comprises BMR, physical activity, and thermogenesis—both obligatory and adaptive. In theory, enhancing TEE through increased physical activity or activation of thermogenesis (e.g., via cold exposure or dietary stimuli) could induce a negative energy balance, promoting weight loss if not offset by compensatory increases in food intake (Fig. 3). This concept was historically exemplified by the weight-reducing effects of the mitochondrial uncoupler DNP, although its clinical use was discontinued due to severe toxicity^59^.

However, studies of cold exposure in rodents have yielded mixed results regarding its effects on BW. While some studies report weight loss, others show no change or even weight gain, likely due to compensatory hyperphagia in response to increased EE^5,60–64^. This suggests that mice defend their BW by increasing food intake under cold-induced EE. The most compelling evidence for the therapeutic potential of BAT in obesity and diabetes, however, lies in its role in DIT. Nearly 50 years ago, Rothwell and Stock observed that rats chronically exposed to a cafeteria diet gained less weight than expected based on caloric intake^65^. This resistance to weight gain was accompanied by increased EE, enlarged BAT depots, and enhanced responsiveness to norepinephrine, pointing to a physiological role for BAT in regulating metabolic efficiency and protecting against obesity. Importantly, this increase in EE could not be attributed to the thermic effect of food (i.e., obligatory thermogenesis), since the differences in EE persisted even when the cafeteria diet was withdrawn and the animals became hypophagic, and was also evident in animals consuming equal amounts of food as chow-fed controls after long-term high-fat feeding^65,66^. These findings indicate that, like CIT, DIT is facultative and adaptive.

### Evolutionary perspective on BAT function

The involvement of BAT in DIT—and even the existence of DIT itself—has been however a subject of ongoing debate^67^. From an evolutionary standpoint, it is reasonable to argue that BAT did not evolve primarily to burn excess calories, especially considering that early humans lived in environments characterized by limited food availability and low rates of obesity. Nonetheless, selective pressure against excessive adiposity—due to its detrimental effects on mobility and increased vulnerability to predation—may help explain why BAT remains sensitive to calorie intake, and the regulation of adaptive thermogenesis by diet and nutrient availability is well documented. For instance, starvation and caloric restriction reduce BMR by approximately 40% and 10%, respectively, reflecting an energy-conserving response during periods of food scarcity. Notably, the reduction in BMR during prolonged starvation exceeds what would be predicted from lean mass loss alone, underscoring the active, adaptive nature of this physiological response^68,69^. While the evolutionary advantage of an energy-dissipating response to feeding may be less obvious than that of energy conservation during fasting, feeding does induce measurable increases in EE—both acutely, via the thermic effect of food, and chronically, with growing evidence implicating BAT in this process.

An evolutionary advantage of increased thermogenesis in response to food intake may lie in the disposal of surplus non-essential energy, particularly when consuming unbalanced or nutrient-poor diets. This is particularly evident with low-protein (LP) diets, which are well known to elevate EE^70^. To obtain adequate protein for growth and amino acids for protein synthesis, animals consuming LP diets must increase their overall food intake—an evolutionarily disadvantageous situation unless excess calories can be dissipated. Early studies in rats revealed that diets containing more than 20% protein elevated thermogenesis, likely due to the high metabolic cost of protein metabolism (i.e., obligatory thermogenesis). Interestingly, diets with protein levels below 20% also increased thermogenesis, suggesting an additional adaptive mechanism beyond obligatory protein metabolism. This was confirmed in piglet studies, where animals on LP diets required nearly five times more energy to maintain the same BW as those on normal-protein (NP) diets^71^. Without such an energy-dissipating mechanism, LP-fed piglets would have accumulated fat in proportion to their increased caloric intake. Further investigations revealed that LP-fed rats displayed increased sensitivity to noradrenaline-induced thermogenesis, a response characteristic of cold-adapted animals, suggesting shared mechanisms between CIT and DIT. Subsequent studies confirmed BAT’s involvement in noradrenaline-stimulated DIT, demonstrating enhanced mitochondrial uncoupling in BAT of rats fed a cafeteria-style LP diet compared to those on high-protein regimens^65,72^.

Observations from nature further support a role for BAT in DIT, particularly in species adapted to LP diets. The fruit bat, for instance, consumes a diet containing only 2–5% protein yet exhibits high energy intake and possesses substantial BAT depots. Similarly, the marmoset—another frugivorous species—displays a robust thermogenic response to noradrenaline and has highly active BAT compared to rats of similar body size. Notably, both species inhabit tropical environments, indicating that BAT function in these animals is unlikely to be primarily related to cold adaptation. Instead, these examples suggest a specialized role for BAT in DIT under conditions of nutrient imbalance or protein scarcity^73,74^.

### BAT in humans

While BAT’s role as a thermoregulatory organ is well established in small rodents and human infants, its significance in adult humans’ metabolic health has only recently gained recognition—particularly in the context of DIT, and metabolically active BAT is now acknowledged as a contributor to energy homeostasis in humans. The first anatomical description of human BAT (hBAT) dates back nearly a century, with subsequent studies characterizing its distribution and morphological features across different life stages. Early functional evidence for BAT activity in humans emerged from studies of outdoor workers exposed to cold environments, who exhibited increased mitochondrial enzyme activity in BAT depots compared to indoor workers^75–77^.

Today, functional BAT in humans is routinely assessed using 2-deoxy-2-[^18^F]fluoro-D-glucose (^18^FDG) as a tracer in positron emission tomography/computed tomography (PET/CT) scans. This technique leverages two key characteristics of BAT: its high ^18^FDG uptake relative to WAT, and its CT radiodensity consistent with adipose tissue. Studies using PET/CT have revealed that hBAT is primarily located in the supraclavicular, paravertebral, pericardial, and suprarenal regions. These depots are activated by cold exposure, and their activation is reduced in individuals with obesity compared to normal weight subjects; moreover, BAT glucose uptake has been positively correlated with metabolic rate, and both BAT activity and mass are inversely associated with BMI^78–80^. However, a consistent link between hBAT activity and CIT in humans remains elusive, likely because skeletal muscle contributes more substantially to CIT^79,81^. As a result, the extent to which hBAT contributes to CIT has been frequently questioned. Experimental findings have been mixed: while some studies report a positive correlation between hBAT activity and CIT under both acute and chronic cold exposure^82–84^, others support a more predominant thermogenic role for skeletal muscle^85,86^. This divergence may help explain why attempts to induce weight loss through cold-mediated BAT activation have generally produced limited success^87–90^, although some exceptions have been reported^91^.

Nonetheless, accumulating evidence suggests that hBAT may contribute to EE in humans. Individuals with obesity exhibit reduced basal BAT activity and volume compared to their lean counterparts, along with diminished ^18^FDG uptake in response to cold exposure^79,92–94^. Additionally, dietary factors appear to influence hBAT activity. A single high-calorie, carbohydrate-rich meal has been shown to activate hBAT in lean adults, although to a lesser extent than cold exposure. Interestingly, BAT glucose uptake following a meal is often lower than in the fasted state, potentially due to increased lipid utilization by hBAT after mixed-nutrient intake or competition with insulin-stimulated glucose uptake by skeletal muscle, which could lead to underestimation of BAT activity using ^18^FDG uptake^95,96^. This underestimation is further complicated by skeletal muscle’s high capacity for glucose clearance during both cold exposure and feeding—especially in individuals with insulin resistance, where impaired muscle glucose uptake may obscure relative BAT activity^97^. More accurate assessment techniques—such as combining CT imaging and indirect calorimetry with specialized radiotracers—have shown that a carbohydrate-rich meal can elevate postprandial EE to a level comparable to that induced by cold exposure. In both conditions, BAT oxygen consumption increases significantly relative to room temperature^98^. Moreover, individuals with higher BAT glucose uptake exhibit greater DIT, supporting a link between BAT activity and postprandial EE. However, while BAT activation—whether triggered by cold exposure or pharmacological stimulation with the β_3_-adrenoceptor agonist mirabegron—correlates with CIT, no consistent association has been observed with DIT^99,100^.

Beyond its thermogenesis role, active hBAT exhibits substantial uptake of glucose and FFA, suggesting potential therapeutic relevance. By functioning as a “glucose sink,” activated BAT emerges as a promising target for managing metabolic disorders characterized by impaired glucose homeostasis, including metabolic syndrome and cardiometabolic disease, which frequently co-occur with obesity. A common critique regarding hBAT’s physiological function it’s toward its relatively small volume, constituting approximately 0.1–0.5% of total body mass in humans, in contrast to the 2–5% observed in rodents. Nonetheless, clinical and translational studies have demonstrated that cold-activated hBAT contributes to improving insulin sensitivity and glucose homeostasis. For example, ten days of cold exposure increased insulin sensitivity by more than 40% in patients with T2DM, while mild cold exposure in healthy men resulted in a 20% increase, accompanied by elevated glucose uptake and noradrenaline turnover, independent of changes in insulin secretion^87,90,101^. Additionally, cold exposure has been shown to increase EE, glucose clearance, and insulin sensitivity in individuals with metabolically active BAT^78^.

In interventional studies, mirabegron administration (100 mg/day for four weeks in healthy women, and 50 mg/day in individuals with obesity) led to increases in BAT mass, resting EE, glucose tolerance, and insulin sensitivity^102,103^. Supporting these findings, a large-scale retrospective analysis of 134,529 ^18^FDG PET/CT scans from 52,487 patients revealed that individuals with detectable BAT exhibited significantly lower prevalence of T2DM, dyslipidaemia, coronary artery disease, cerebrovascular disease, heart failure, and hypertension. Notably, the protective association between BAT activity and cardiometabolic health was particularly pronounced in subjects with obesity, suggesting the potential of hBAT activation as a therapeutic strategy for metabolic disease prevention and management^104^.

Although the extent to which glucose serves as a thermogenic substrate in BAT remains a topic of debate, several studies support a functional role for glucose in thermogenesis^97^. Chronic cold exposure and β_3_-adrenergic stimulation have been shown to enhance glucose oxidation in brown adipocytes, while genetic inhibition of the mitochondrial pyruvate carrier disrupts cold-induced glucose oxidation and impairs thermoregulation in mice^105^. β-adrenergic signaling also activates mammalian target of rapamycin 2 (mTORC2), promoting glucose uptake and glycolysis in BAT; notably, mice lacking mTORC2 in adipose tissue exhibit cold intolerance^106^. In addition, optogenetic stimulation of sympathetic nerves innervating BAT lowers plasma glucose levels and increases body temperature—a thermogenic effect that is abrogated by inhibition of lactate dehydrogenase, implicating glycolytic flux^107^. Recent metabolic flux analysis (MFA) by Bornstein and colleagues further outlines substrate usage in BAT under cold stress: in fed mice, glucose is the predominant fuel, whereas in the fasted state, BAT shifts to FA oxidation. Under these conditions, glucose primarily contributes to TCA cycle anaplerosis, potentially supporting biosynthetic demands or fueling futile substrate cycles that drive thermogenesis^108^.

## Mitochondrial uncoupling, glucose metabolism, and cancer

### BAT and cancer

**Fig. 4:**
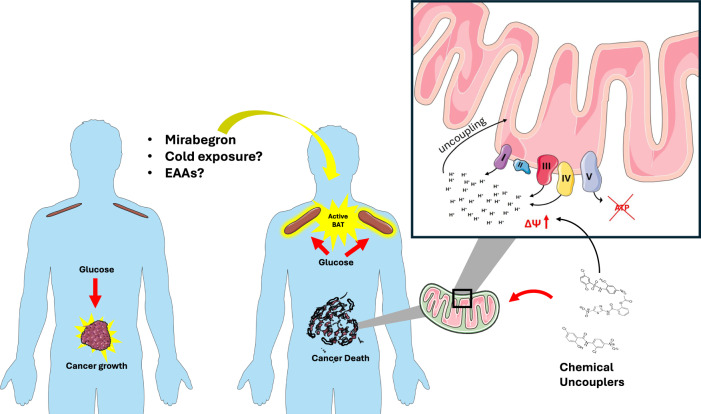
Interplay between BAT, cancer metabolism, and mitochondrial uncoupling. Glucose addiction and high ΔΨ of cancers can be exploited for therapeutic purposes by means of endogenous BAT activation or exogenous uncouplers. Both cancer cells and activated BAT rely on high glucose utilization. Physiological (e.g., cold exposure) or pharmacological (e.g., β_3_-adrenergic agonists) activation of BAT promotes glucose utilization, acting as a metabolic “sink” that deprives cancer cells of glucose, thereby limiting tumor growth. Dietary supplementation with essential amino acids (EAAs), known to stimulate BAT, may also represent a nutritional anticancer strategy. Alternatively, exogenous small-molecule mitochondrial uncouplers have demonstrated anticancer effects by disrupting oxidative phosphorylation. See main text for abbreviations. Image adapted from Servier Medical Art (https://smart.servier.com/), licensed under CC BY 4.0 (https://creativecommons.org/licenses/by/4.0/).

The clinical utility of ^18^F-FDG PET imaging in oncology is based on its ability to detect tissues with high glucose uptake, reflecting the Warburg effect—a metabolic hallmark of cancer whereby cancer cells preferentially rely on glycolysis to support rapid proliferation and biomass accumulation. Intriguingly, PET scans of cancer patients often reveal pronounced ^18^F-FDG uptake in the supraclavicular region, which has been identified as metabolically active BAT. This observation underscores a potential metabolic competition between tumors and BAT for glucose and raises the possibility that BAT activation may reduce systemic glucose availability, thereby limiting tumor growth^109^ (Fig. 4). Preclinical studies support this concept: cold exposure enhances glucose uptake by BAT, reduces circulating glucose levels, and suppresses tumor progression in several cancer models, including breast cancer, pancreatic adenocarcinoma, colorectal cancer, melanoma, hepatocellular cancer, and fibrosarcoma. Importantly, these anticancer effects are abolished in *Ucp1*-deficient mice, indicating that mitochondrial uncoupling is essential for BAT-mediated tumor suppression^5^. Clinical observations also supported these findings; for example, mild cold exposure (22 °C for one week) in a young patient with Hodgkin’s lymphoma was associated with reduced tumor glucose uptake^5^. Similarly, BAT activation with mirabegron inhibited tumor growth in mouse models by diverting glucose uptake toward BAT^6^. Again, this effect was absent in *Ucp1* knockout mice, reinforcing the central role of mitochondrial uncoupling in mediating these outcomes. More recently, BAT activation via cold exposure or β_3_-agonist treatment has been shown to protect against highly glycolytic hematologic malignancies, including acute myeloid leukemia (AML) and acute lymphoblastic leukemia (ALL). These interventions not only slowed disease progression but also potentiated the effects of conventional chemotherapy—an effect entirely dependent on the presence of functional UCP1^110^.

Despite these pre-clinical findings, the translational potential of BAT activation as an anti-cancer strategy in humans remains to be determined. As discussed above, hBAT mass has a relatively low mass compared to that in mice. Although hBAT responds to both pharmacological and environmental stimuli^87,90,102,103^, its amount varies greatly among individuals and is influenced by several anthropometric factors. Most importantly, BAT mass declines with age and obesity^92,111^ —both conditions associated with increased cancer incidence—which may reduce responsiveness to pharmacological, nutritional, or environmental activation in humans.

Additional evidence underscores the requirement of mitochondrial uncoupling for BAT-mediated glycaemic control. Although ^18^F-FDG uptake in BAT following CL316,243 injection appeared similar in *Ucp1* knockout mice and wild-type mice, prolonged β_3_-adrenergic stimulation improved glucose homeostasis only in wild-type animals. This effect was absent in *Ucp1*-deficient mice, indicating that the glucose-lowering benefit of chronic BAT activation is thermogenesis-dependent^112^. Further mechanistic insight reveals that UCP1 is essential for norepinephrine-stimulated, but not insulin-stimulated, glucose uptake. The underlying mechanism involves UCP1-mediated reductions in ATP synthesis, which increase the intracellular AMP/ATP ratio, thereby activating AMP-activated protein kinase (AMPK) and subsequent translocation of type 4 glucose transporter (GLUT4) to the plasma membrane, enhancing glucose uptake in brown adipocytes^113^.

### Mitochondrial uncoupling, UCPs, and cancer

UCP1-mediated glucose metabolism is critical for the anticancer effects observed with both cold exposure and mirabegron treatment^6^. These findings raise an intriguing question: beyond acting as a metabolic sink that limits glucose availability to tumors and improving systemic cardiometabolic health, does mitochondrial uncoupling itself exert direct anticancer effects? To sustain uncontrolled proliferation, cancer cells undergo profound metabolic reprogramming, primarily through two major metabolic adaptations: the Warburg effect and glutaminolysis. The Warburg effect enables cancer cells to favor glycolysis over mitochondrial pyruvate oxidation, thereby diverting glycolytic intermediates into anabolic pathways essential for biomass production. In parallel, glutaminolysis transforms mitochondria from catabolic organelles into biosynthetic hubs that generate nucleotides, amino acids, and lipids^114,115^. Consequently, interventions that enhance mitochondrial oxidative metabolism, such as cold- or diet-induced mitochondrial uncoupling in BAT, could theoretically counteract these metabolic programs. By promoting mitochondrial catabolism and limiting the diversion of substrates toward anabolic pathways, mitochondrial uncoupling may directly interfere with the metabolic flexibility of cancer cells, thereby restraining tumor progression^58,116^.

While mitochondrial uncoupling through UCP1 in BAT appears to exert anticancer effects, early evidence suggests a more complex and context-dependent relationship between mitochondrial proton leak and tumor biology. In some cancers, uncoupling may actually confer a growth advantage. For instance, drug-resistant cancer cell lines display lower ΔΨ, increased proton leak, and elevated expression of UCP2 compared to drug-sensitive counterparts^117^. These adaptations are associated with reduced ROS production, enhanced DNA repair capacity, and greater metabolic flexibility—particularly an increased ability to utilize FA when glucose availability is limited. These findings support the hypothesis that mitochondrial uncoupling may exert an antioxidant function that promotes cancer cell survival under metabolic stress^117^. Studies investigating the expression and function of UCP1 and its homologs (UCP2 and UCP3) in various tumor types have yielded heterogeneous results. UCPs have often been associated with tumor growth, although notable exceptions exist. UCP1, for example, is ectopically expressed in prostate cancer and is upregulated in non-small cell lung cancer (NSCLC) and colorectal carcinoma^118,119^. Conversely, UCP1 expression is reduced in breast cancer and positively associated with patient survival. Specifically, in triple-negative breast cancer (TNBC), UCP1 levels are significantly lower than in normal tissue, and its overexpression suppresses tumor growth both in vitro and in vivo^120^. Furthermore, UCP1 is more highly expressed in normal mammary epithelium and well-differentiated breast tumors than in poorly differentiated, high-grade tumors, and elevated UCP1 expression is associated with a favorable prognosis^121^. These findings suggest that UCP1 may exert tumor-suppressive functions in breast cancer. However, interpreting these data requires consideration of the unique metabolic and cellular composition of the breast microenvironment, which is rich in adipose tissue—including both WAT and BAT depots. Adipose tissue contributes to breast tumorigenesis through the secretion of pro-inflammatory cytokines and other paracrine signals, particularly in the context of obesity^122^. As such, UCP1 expression in breast tumors likely reflects not only intrinsic cancer cell metabolism, but also the individual’s systemic metabolic state, the extent of BAT activity, and the degree of WAT browning within the breast tissue microenvironment^123,124^.

It is worth noting that UCP1 is the only UCP definitely confirmed to mediate thermogenesis and directly regulate EE^125,126^. Consequently, the anticancer effects associated with UCP1 activation in BAT—via systemic glucose depletion and cardiometabolic improvements—may involve mechanisms that are fundamentally distinct from those through which other UCP homologs modulate cancer metabolism. Unlike UCP1, which is restricted primarily to brown and beige adipocytes, other isoforms have broader tissue distribution and physiological roles. UCP2, for instance, is widely expressed and primarily involved in the regulation of mitochondrial ROS levels, while UCP3 is more closely linked to lipid metabolism and is predominantly expressed in skeletal muscle and brown fat. These differences underscore the need to consider the tissue-specific context and functional specialization of each UCP isoform when evaluating their roles in cancer biology.

These distinctions among UCP isoforms suggest that uncoupling OXPHOS could, under certain conditions, promote tumor progression through mechanisms distinct from UCP1-mediated glucose competition observed in BAT. By reducing mitochondrial ATP synthesis and limiting its export to the cytosol, uncoupling may relieve the allosteric inhibition of phosphofructokinase (PFK)—the rate-limiting enzyme in glycolysis that is inhibited by high cytosolic ATP levels, thus enhancing glycolytic flux and supporting the metabolic demands of rapidly proliferating cancer cells^127^. Alternatively, uncoupling may influence tumor growth by regulating mitochondrial ROS production. UCPs are known to be activated by superoxide anions, and pharmacologic inhibition of UCPs (e.g., with GDP) has been observed to increase mitochondrial hydrogen peroxide levels^128,129^. These findings support the hypothesis that UCP-mediated proton leak may exert an antioxidant function, potentially protecting cancer cells from oxidative stress and contributing to therapy resistance.

The discovery that UCP2-deficient mice resist to *Toxoplasma gondii* infection, attributed to elevated ROS production in macrophages^130^, has brought renewed attention to UCP2’s role in modulating immune and metabolic responses, with important implications for cancer biology. UCP2 is frequently overexpressed in a range of malignancies, including breast cancer, leukemia, colon cancer, and thyroid tumors. Elevated UCP2 expression has been linked to increased tumor growth, chemoresistance, and promotion of the Warburg effect. In contrast, *Ucp2* knockout restores sensitivity to chemotherapeutic agents and reverses metabolic reprogramming, shifting cells away from the Warburg effect^131,132^. Mechanistically, UCP2 overexpression enhances glucose utilization into amino acids, nucleotides, and TCA cycle intermediates^133^. UCP2 also facilitates glutaminolysis by functioning as a mitochondrial protons/C4-metabolite exchanger and by exporting cytosolic aspartate for NADPH synthesis^134,135^. These metabolic shifts underscore UCP2’s role in reprogramming mitochondrial function to favor anabolic processes over ATP production. However, UCP2’s role in cancer appears context-dependent, with evidence also tumor-suppressive functions. In some models, UCP2 induction enhances antitumor immune responses within the tumor microenvironment^136^. Moreover, UCP2 overexpression has been associated to reduced cancer cell proliferation and a metabolic shift toward oxidative phosphorylation, both in vitro and in vivo^137^. Supporting its potential protective role, *Ucp2*-deficient mice treated with the carcinogen azoxymethane develop more colon tumors than wild-type controls and exhibit increased oxidative stress—consistent with UCP2’s function as a mitochondrial ROS scavenger^138^. These findings highlight UCP2’s dual role in cancer, which appears context-dependent and influenced by factors such as tissue type, metabolic conditions, and experimental models. It is important to note that UCP2 is subject to strong translational regulation, meaning that mRNA levels do not always correlate with protein expression. Additionally, overexpression models may introduce technical artifacts or trigger compensatory upregulation of other UCP isoforms, complicating interpretation of experimental results^139–141^.

In contrast, the role of UCP3 in cancer remains poorly defined. UCP3 is primarily expressed in muscle, heart, and BAT, and its expression is paradoxically induced during fasting. These observations suggest that UCP3 plays a specialized role in lipid metabolism, particularly during transitions between fed and fasted states or during physical activity, rather than acting as a major regulator of EE or tumor metabolism^142^. Furthermore, UCPs activity is modulated by several endogenous cofactors—including coenzyme Q, superoxide anions, and purine nucleotides—which can influence their physiological and pathological roles^142^. These regulatory factors should be taken into account when evaluating UCP function—particularly that of UCP2 and UCP3. Interpreting changes in their expression as a proxy for activity should therefore be done with caution. Overall, the actual roles of UCP2 and UCP3 in both physiological and pathological contexts remain largely unresolved^143^.

Table 1 summarizes the expression patterns of UCPs in human cancers, the detection methods used, and—given that UCP expression does not necessarily reflect functional activity^143^ —any functional assays that were performed.

**Table 1:** Endogenous expression of UCPs in human cancers

UCP1	CANCER TYPE	REGULATION	METHODS OF DETECTION	ASSAY ON MITOCHONDRIAL/UNCOUPLED RESPIRATION	REFERENCE
	Prostate cancer	Upregulated In clinical specimen	IHC	no	^118^
	Triple-negative breast cancer	Downregulated in bt549 cell line and specimen	WB and IHC	no	^120^
	Non-small cell lung cancer (NSCLC - alveolar cells)	Upregulated in specimen	IHC	No	^119^
	Breast cancer	Downregulated in clinical specimen	IHC	no	^176^
	Colorectal carcinoma	Upregulated In specimen	IHC	no	^177^
	Myeloid-derived suppressor cells	Downregulated	WB	no	^178^
	ccRCC	Downregulated	WB and IHC	no	^179^
	Breast cancer	Upregulated in ER positive and differentiated breast tumors	IHC	Yes increase in proton leak in vitro	^121^
	Breast cancer	Expression positively associated with prognostic status and RFS/OS	IHC	thermal tomography; higher expression in patients with q-r curves at 30 °–45 °	^180^
**UCP2**	Cancer Cell lines	Upregulated in resistant vs sensitive	WB	Yes (increase in OCR together with decrease in MMP) in vitro	^117^
	Colon cancer	upregulated	IHC and WB	no	^181^
	Colon cancer	upregulated	IHC WB	no	^182^
	Breast Cancer	Upregulated in grade 3 vs grade 1	IHC and WB	Yes in primary tumor cells Higher MMP in grade 3 vs grade1 cells in vitro	^183^
	Breast ovarian, bladder, esophageal, testicular, kidney, colorectal, lung, pancreas prostate leukemia	Upregulated	Microarray	In vitro overexpression - decrease in MMP	^184^
	Head and neck, skin, prostate, and pancreatic tumor samples	Upregulated vs surrounding normal tissues	WB	no	^185^
	Patient-derived ovarian cancer stem cells	Upregulated vs differentiated	WB	Yes (higher MMP)	^186^
	clear cell renal cell carcinoma (ccRCC)	Downregulated In patient tumor samples vs surrounding normal tissues	WB	Yes on isolated mitochondria from ccRCC samples (higher MMP in tumor samples)	^187^
	uterine cervical cancer	Expression positively related with sensitivity to neo adjuvant chemotherapy and negatively with survival	IHC	No	^188^
	Ovarian carcinoma	Expression positively related with sensitivity to platinum and negatively with survival	IHC	No	^189^
	Melanoma	Upregulated in patients with high T cell anti-tumor responses	Single cell RNAseq	no	^136^
	Colorectal cancer	Upregulated vs normal mucosa samples	WB	In vitro UCP2 KO in tumors show increase in glycolysis with no effect on OXPHOS or proton leak	^190^
	Gallbladder cancer (GBC)	Upregulated vs surrounding normal tissues	WB	In vitro Inhibition on GBC results in increase in MMP	^191^
	Uterine cervical cancer	Expression positively related with clinical stage and lymph node metastasis	IHC	no	^192^
	Breast cancer	Expression positively associated with prognostic status and RFS/OS	IHC	thermal tomography; no change between q-r curves in patients	^180^
**UCP3**	NSCLC	Upregulated In specimen	IHC	no	^119^
	ccRCC	Upregulated	WB	no	^193^
	ccRCC	Upregulated In patient tumor samples vs surrounding normal tissues (only the long isoform UCP3L)	WB	Yes on isolated mitochondria from ccRCC samples(higher MMP in tumor samples)	^187^

*WB* western Blot, *IHC* immunohistochemistry.

### Chemical uncouplers and cancer

In striking contrast to the context-dependent effects of endogenous UCPs, exogenous chemical uncouplers have demonstrated consistent anticancer activity in both in vitro and in vivo models.

Since the discontinuation of DNP, efforts have focused on developing safer uncouplers with broader therapeutic windows, particularly for obesity and diabetes. Several of these newer uncouplers have demonstrated efficacy in reducing tumor growth in vivo^7^. Niclosamide, an FDA-approved antiprotozoal drug, has demonstrated potent antitumor activity, both as a monotherapy and in combination with irinotecan, in colorectal, glioblastoma, and breast cancer models^144–146^; Nitazoxanide, another FDA-approved antiprotozoal drug, exhibits similar efficacy against the same tumor types, including enhancement of irinotecan activity in HCT116 colorectal cancer xenografts^147–149^. FH535, originally identified as an inhibitor of Wnt/β-catenin and PPAR signaling, also functions as a mitochondrial uncoupler and suppresses hepatocellular carcinoma and colon cancer growth, particularly when combined with sorafenib^150–152^. SR4, another uncoupling agent, has been shown to suppress melanoma and lung cancer growth in vivo^153,154^. Importantly, these compounds have not been associated with the severe toxicities typical of earlier uncouplers. While nitazoxanide treatment was associated with a modest 7–10% reduction in BW, none of the studies reported hyperthermia or other adverse events characteristic of classical uncoupling toxicity^149^. These findings suggest that the anticancer effects of chemical uncouplers are mechanistically distinct from those mediated by UCP1 activation in BAT. Notably, their efficacy appears to be independent of increased whole-body EE, implicating alternative mechanisms—such as mitochondrial metabolic stress or inhibition of biosynthetic pathways—as drivers of tumor suppression (Fig. 4).

The compelling evidence that both exogenous mitochondrial uncouplers and UCP1 activation in BAT impair tumor growth presents a paradox when compared with the frequent upregulation of endogenous UCPs—particularly UCP2—in human cancers. In the case of BAT activation, the underlying mechanism appears relatively straightforward: enhanced glucose uptake by thermogenically active BAT reduces systemic glucose availability, thereby limiting a key fuel source for tumors. However, the role of endogenous UCPs in cancer is more complex and some aspects must be taken into account when analyzing this apparent contradiction; elevated expression or activity of UCPs—especially UCP2—in cancer cells may be selected during tumor development as an adaptive mechanism. By reducing mitochondrial ROS production optimizing substrate flexibility, and enhancing glycolysis, UCP2 overexpression can confer a survival advantage in the context of metabolic and oxidative stress^127,133,134^.On the other hand, cancer cell mitochondria are known to be hyperpolarized—with membrane potentials averaging ~−220 mV, compared to ~−140 mV in non-transformed cells^155–157^. This bioenergetic adaptation renders therefore cancer cells particularly susceptible to exogenous uncouplers, In this context, both hyperpolarization and controlled depolarization (via endogenous UCP expression) can paradoxically benefit cancer cells: the former supports bioenergetic capacity and ROS signaling, while the latter mitigates ROS toxicity and preserves redox balance. These seemingly conflicting results highlight the extraordinary metabolic plasticity of cancer cells, which are able to adapt to diverse microenvironmental and energetic conditions. Indeed, elevated mitochondrial ∆Ψ can drive ROS accumulation, whereas mild uncoupling via UCPs can serve to buffer oxidative stress. This duality is consistent with a growing body of evidence showing that ROS can act as both tumor-promoting and tumor-suppressing agents, depending on dose, context, and localization^158^.

It should also be kept in mind that, when considering the potential anti-cancer therapeutic benefits of either exogenous uncoupling or brown fat activation, the associated increase in EE may have detrimental effects in the context of cachexia—a common and severe cancer-related complication characterized by a hypermetabolic state, weight loss, and reductions in both skeletal muscle and fat mass^159^. In mice, evidence of enhanced sympathetic stimulation of BAT in cancer cachexia (CC) was reported as early as 1981^160^. More recent studies have shown that CC is associated with dysregulated BAT activation and increased thermogenesis even under thermoneutral conditions. This contributes to inflammation and anorexia, along with WAT browning, elevated lipolysis, increased EE, inflammatory cytokine release, and muscle wasting in cachectic mice^161,162^. With regard to human studies, early reports showed that children with various malignancies displayed increased GDP-binding capacity in BAT mitochondria—a proxy of UCP1 activity—compared to non-malignant controls. In addition, light microscopy of peri-adrenal fat from post-mortem examinations revealed the presence of BAT in 80% of cachectic cancer patients^163,164^. More recently, the use of advanced imaging techniques such as ^18^F-FDG-PET-CT has enabled a more refined investigation of this issue. Using this technique in retrospective studies, some reports identified a positive correlation between BAT activity and cancer presence (but not CC)^165–168^. However, none of these studies accounted for outdoor temperature, a variable that could significantly influence BAT activation and thus confound the relationship between BAT activity and disease status or progression. When temperature was controlled for, one retrospective study found no association between BAT activity and either CC or cancer-related mortality^169^. Similarly, a large retrospective longitudinal FDG-PET/CT analysis involving 8,409 patients found no significant association between BAT and CC^170^. Noteworthy, a recent retrospective cohort study—carefully matched for anthropometric and disease-related characteristics, season, and outdoor temperature at the time of PET analysis—reported that patients with larger BAT depots at cancer diagnosis had a lower risk of developing weight loss and cachexia compared to BAT-negative patients^171^. Overall, most retrospective ^18^F-FDG-PET/CT studies on BAT and cancer in humans lack corrections for key confounding factors that modulate BAT activity, including diet, cold exposure, physical activity, and body mass index. These limitations should be carefully considered when interpreting their findings^172^.

Clinical treatment of CC currently involves both pharmacological and nutritional strategies. Several anti-inflammatory, anabolic, and orexigenic drugs have been tested in clinical trials, with mixed outcomes^173^. Notably, a recent trial involving a monoclonal antibody targeting GDF15 (growth differentiation factor 15)—a cytokine elevated in cachectic patients—has shown promising results^174^. Among non-pharmacological interventions, high-protein diets, essential amino acid (EAA) supplementation, and other nutritional approaches have been proposed to mitigate cachexia-induced muscle wasting^175^. Interestingly, EAA supplementation has also been shown in mice to impair tumor growth and to activate BAT thermogenesis, leading to reduced BW and fat mass while preserving lean mass^56,58^. These findings suggest that EAA supplementation may represent a safe and effective strategy to counteract potential muscle loss resulting from BAT activation within an anti-cancer therapeutic framework.

Overall, although preclinical evidence clearly supports a potential role for BAT in exacerbating cancer-related complications, including cachexia, the association between BAT and CC in humans remains insufficiently explored and warrants further investigation under more standardized and controlled conditions.

## Discussion

Mitochondrial uncoupling of OXPHOS plays a central role in regulating mammalian metabolism, with extensive implications for both physiological and pathological processes. While the thermogenic effects of uncoupling—particularly in CIT and DIT—are well recognized in animal models, their full significance in humans remains under active investigation. In particular, while CIT has a clearly established physiological and evolutionary function, the existence and relevance of DIT are still debated. BAT, a specialized thermogenic organ, contributes to both CIT and DIT in rodents, and accumulating evidence suggests that it plays an important role in glucose homeostasis in humans. Intriguingly, activated BAT can also work as a metabolic “glucose sink,” reducing systemic glucose availability and exerting antitumor effects by limiting the energy supply to tumors. Although human data remain inconclusive, pharmacological uncoupling of OXPHOS has consistently demonstrated anticancer effects in preclinical models, independent of thermogenesis. Despite these promising findings, significant knowledge gaps persist regarding the roles of endogenous UCPs in human cancers. Most of the existing studies are correlative, focusing on differential UCP expression between tumor and normal tissues and functional analyses of uncoupled respiration in cancer cells—and comparisons with adjacent normal cells—are rare but needed (see Table 1). Direct evaluations of how UCP upregulation affects mitochondrial function, ROS dynamics, and metabolic reprogramming in the tumor microenvironment are essential to clarify these contradictory findings. BAT represents a metabolically dynamic and nutritionally responsive tissue, capable of deeply altering systemic metabolism upon activation. As such, BAT’s emerging role in diseases characterized by metabolic dysfunction—including obesity, diabetes, and cancer—holds therapeutic promise. Combining BAT-activating agents or mitochondrial uncouplers with dietary interventions could offer novel strategies to improve cardiometabolic health and combat cancer by reshaping the host’s metabolic environment.

## Data Availability

No datasets were generated or analyzed during the current study.
